# Supplementary material for: A UMLS-based spell checker for natural language processing in vaccine safety
Source: BMC Med Inform Decis Mak. 2007 Feb 12;7:3. doi: 10.1186/1472-6947-7-3 (PMC1805499; doi:10.1186/1472-6947-7-3)
Supplement: Additional file 13 — RAP application directory. Sets up directory for RAP (RDF application for PHP) source code [file 1472-6947-7-3-S13.gz › rap/doc/phpdoc/dataset/Quad.html]

xml version="1.0" encoding="iso-8859-1"?


Docs For Class Quad


## Class Quad

Description

Description |
Methods (details)

A Triple in a RDF dataset, consisting of four Jena Nodes: graphName, subject, predicate, and object.

History:

- access: public
- author: Daniel Westphal (http://www.d-westphal.de)
- version: V0.9

Located in /dataset/Quad.php (line 20)

Method Summary

Description |
Methods (details)

Quad
Quad
(Resource $graphName, Resource $subject, Resource $predicate, Resource $object)

boolean
equals
(Quad $quad)

Resource
getGraphName
()

Resource
getObject
()

Resource
getPredicate
()

statement
getStatement
()

Resource
getSubject
()

void
setGraphName
(Resource $graphName)

string
toString
()

Methods

Description |
Methods (details)

Constructor Quad (line 49)

Constructor Creates a Quad from four Nodes.

- access: public

Quad

Quad
(Resource $graphName, Resource $subject, Resource $predicate, Resource $object)

- Resource
  $graphName
- Resource
  $subject
- Resource
  $predicate
- Resource
  $object

equals (line 144)

Checks, if two quads are equal.

- access: public

boolean

equals
(Quad $quad)

- Quad
  $quad

getGraphName (line 78)

Return the graph name.

- access: public

Resource

getGraphName
()

getObject (line 121)

Return the object.

- access: public

Resource

getObject
()

getPredicate (line 110)

Return the predicate.

- access: public

Resource

getPredicate
()

getStatement (line 132)

Return the statement(subject,predicate,object).

- access: public

statement

getStatement
()

getSubject (line 99)

Return the subject.

- access: public

Resource

getSubject
()

setGraphName (line 67)

Sets the graph name.

- access: public

void

setGraphName
(Resource $graphName)

- Resource
  $graphName

toString (line 88)

Return a human-readable (sort of) string "graphname { s p o . }" describing the quad.

string

toString
()

Documentation generated on Thu, 7 Jul 2005 13:42:38 +0200 by phpDocumentor 1.3.0RC3
